# Differences in perceptual masking between humans and rats

**DOI:** 10.1002/brb3.1368

**Published:** 2019-08-24

**Authors:** Katrina L. Dell, Ehsan Arabzadeh, Nicholas S. C. Price

**Affiliations:** ^1^ Neuroscience Program, Biomedicine Discovery Institute Monash University Clayton Vic. Australia; ^2^ Department of Physiology Monash University Clayton Vic. Australia; ^3^ Australian Research Council Centre of Excellence for Integrative Brain Function Monash University Node Clayton Vic. Australia; ^4^ Department of Medicine, St. Vincent's Hospital The University of Melbourne Fitzroy Vic. Australia; ^5^ John Curtin School of Medical Research, Eccles Institute of Neuroscience The Australian National University Canberra ACT Australia; ^6^ Australian Research Council Centre of Excellence for Integrative Brain Function The Australian National University Node Canberra ACT Australia

**Keywords:** backward masking, orientation detection, perception, rat

## Abstract

**Introduction:**

The perception of a target stimulus can be impaired by a subsequent mask stimulus, even if they do not overlap temporally or spatially. This “backward masking” is commonly used to modulate a subject's awareness of a target and to characterize the temporal dynamics of vision. Masking is most apparent with brief, low‐contrast targets, making detection difficult even in the absence of a mask. Although necessary to investigate the underlying neural mechanisms, evaluating masking phenomena in animal models is particularly challenging, as the task structure and critical stimulus features to be attended must be learned incrementally through rewards and feedback. Despite the increasing popularity of rodents in vision research, it is unclear if they are susceptible to masking illusions.

**Methods:**

We characterized how spatially surrounding masks affected the detection of sine‐wave grating targets.

**Results:**

In humans (*n* = 5) and rats (*n* = 7), target detection improved with contrast and was reduced by the presence of a mask. After controlling for biases to respond induced by the presence of the mask, a clear reduction in detectability was caused by masks. This reduction was evident when data were averaged across all animals, but was only individually significant in three animals.

**Conclusions:**

While perceptual masking occurs in rats, it may be difficult to observe consistently in individual animals because the complexity of the requisite task pushes the limits of their behavioral capabilities. We suggest methods to ensure that masking, and similarly subtle effects, can be reliably characterized in future experiments.

## 1 INTRODUCTION

The perception of a visual stimulus is not solely determined by the properties of the stimulus itself but also by its temporal and spatial surroundings. For example, in backward visual masking, the neuronal representation and perception of a brief target stimulus (typically 10–100 ms) can be reduced by a mask stimulus that appears after the target (Breitmeyer, 2008). Importantly, these deficits can occur even when the mask does not overlap the target in space or time, meaning that perception of the target is altered without changing its physical properties. Visual masking therefore offers a powerful tool to investigate the neuronal correlates of perception. The neuronal mechanisms responsible for visual masking are unclear, but likely involve a complex interaction of mechanisms occurring throughout the retina, thalamus and cortex (Alwis, Richards, & Price, 2016; Fehmi, Adkins, & Lindsley, 1969; Levick & Zacks, 1970; Rolls, Tovee, & Panzeri, 1999).

In visual masking, the effect of the mask on target perception is usually measured as a change in the ability to detect or discriminate the target. In general, the presence of a mask will impair target perception; however, the magnitude of this effect depends on a range of stimulus properties including target and mask contrast, duration, size, spatial overlap, spatial separation, and temporal separation (stimulus onset asynchrony; Alpern, 1953; Breitmeyer, 1978; Breitmeyer & Ogmen, 2006; Growney, Weisstein, & Cox, 1977; Saarela & Herzog, 2009). For example, the ability to correctly report the presence of a small target grating is reduced if a second, surrounding mask (i.e., an annulus) is presented after the target disappears (Saarela & Herzog, 2008). The observer's ability to detect the target can be improved if the contrast of the target is increased or further impaired if the contrast of the mask is increased (Saarela & Herzog, 2008, 2009). Although the effect of stimulus contrast in visual masking has been clearly defined in humans, it has never been characterized in an animal model.

To investigate the neuronal mechanisms of visual masking, an animal model is necessary. Yet, few studies have collected neuronal responses to visual masking stimuli (Bridgeman, 1975, 1980; Coenen & Eijkman, 1972; Fehmi et al., 1969; Kovács, Vogels, & Orban, 1995; Levick & Zacks, 1970; Macknik & Livingstone, 1998; Macknik & Martinez‐Conde, 2004; Rolls & Tovee, 1994; Rolls et al., 1999; Schiller, 1968; Schiller & Chorover, 1966; Schwartz & Pritchard, 1981; Vaughan & Silverstein, 1968), and even fewer have collected perceptual data in the same species (Bridgeman, 1980; Fehmi et al., 1969; Kovács et al., 1995; Macknik & Livingstone, 1998). This is necessary to confirm that any changes observed in neuronal activity coincide with perceptual deficits. Recently, rodents have become a popular choice for vision research (Juavinett & Callaway, 2015; Lee et al., 2012; Reinagel, 2014). In particular, they provide an expansive range of sophisticated genetic tools to monitor and manipulate specific neuronal subsets and circuits (Huberman & Niell, 2011; Juavinett & Callaway, 2015; Lee et al., 2012). Although rodents possess a visual system that is less specialized and differentiated than that of nonhuman primates, with lower spatial acuity and contrast sensitivity (Busse et al., 2011; Histed, Carvalho, & Maunsell, 2012; Prusky, Harker, Douglas, & Whishaw, 2002), it is clear that they are capable of learning and performing visual tasks with performance levels comparable to that of nonhuman primates (Bossens & Op de Beeck, 2016; Busse et al., 2011; Histed et al., 2012; Meier, Flister, & Reinagel, 2011; Tafazoli, Di Filippo, & Zoccolan, 2012; Zoccolan, 2015). For these reasons, we sought to determine if the rat was a suitable model for research in visual masking and perception.

In an electrophysiological investigation of visual masking in anaesthetized Long Evans rats, we showed that in primary visual cortex, neuronal responses to oriented circular gratings were altered by the presentation of a mask with analogous trends to those observed in other mammalian species (Alwis et al., 2016; Bridgeman, 1975, 1980; Kovács et al., 1995; Rolls et al., 1999). Population decoding of these data allowed us to reliably predict orientation in a coarse discrimination task (e.g., horizontal vs. vertical), and decoding performance decreased when a mask was presented (Dell, Arabzadeh, & Price, 2018). However, in well‐trained animals, no effects of masking were observed, presumably because the conditions that best allow masking to be observed (i.e., short stimulus durations and low contrasts) led to near‐threshold discrimination performance.

Here we seek to determine if perceptual deficits can be observed in Long Evans rats performing a detection task. We first characterized the effects of visual masking in humans performing a target detection task where we varied target contrast, SOA, and the spatial separation between stimuli. The mask impaired target detection across all target contrasts with the greatest effects of the mask occurring at an SOA of 50 ms. We subsequently trained rats to perform a two‐interval forced choice detection task, in which they were rewarded for reporting the interval that contained a target grating. Targets varied in contrast and were followed by a mask (SOA = 50 ms) on some trials. As in the human subjects, target detectability decreased with target contrast. However, the presentation of a mask, which on its own was uninformative, biased all rats (but not humans) to respond more frequently, regardless of whether the target was low contrast or absent. When we controlled for this response bias, the influence of the mask on target detectability was evident when responses were averaged across all animals. Altogether, our results suggest that while perceptual masking occurs in rats, it may be difficult to observe consistently in individual animals because the complexity of the requisite task is at the limits of their perceptual and behavioral capabilities. We suggest methods to ensure that masking can be reliably characterized in future experiments.

## 2 METHODS

### 2.1 Ethics

All experimental procedures involving animals were approved by the Monash University Committee for Ethics in Animal Experimentation (MARP2015/003) and were conducted in accordance with the National Health and Medical Research Council guidelines for the care and welfare of experimental animals, and the ARVO Animal Statement. All experimental procedures involving humans were approved by the Monash University Human Research Ethics Committee (CF16/392 ‐ 2016000178) and were conducted in accordance with the National Statement on Ethical Conduct in Human Research.

### 2.2 Human perception

Two authors and three naïve subjects took part in the experiment. All subjects had normal or corrected to normal vision. Each subject performed a training session prior to data collection.

Target stimuli were sine‐wave gratings with orientation 0 or 90°, spatial frequency 3 cpd, and limited to a circular annulus with diameter 8°. Mask stimuli were plaids (0 + 90°) presented in annuli with an outer diameter of 15.5° and an inner diameter that either matched the 8° target, or was 14.5°, providing 3.25° separation between target and mask. Target stimuli were presented for 23.5 ms and appeared on 50% of trials. Mask stimuli were presented for 100 ms at three stimulus onset asynchronies (0, 50 and 100 ms) relative to the target. A mask stimulus was presented in 66% of trials, with equal probability of the 8 or 14.5° inner diameter mask being shown. Target contrast was varied between 1% and 32% while mask contrast was always 100%. In total, there were 63 conditions (zeros plus six nonzero target contrasts; three SOAs; three mask conditions).

All stimuli were generated using Psychtoolbox in MATLAB and were presented on an 85 Hz refresh rate CRT monitor positioned at a viewing distance of 50 cm. Head position was stabilized with a chin rest. Responses to 1,728 trials/subject were collected across two sessions. Within a session, trials were presented in blocks of 50 allowing participants to take frequent breaks. At the beginning of each trial, participants fixated on a small cross, located 5° to the left of screen center. The target and mask stimuli were presented 5° to the right of the screen center. Following stimulus presentation, the participants were required to indicate whether they had perceived a target stimulus by button press. Correct detections were indicated by a brief tone.

### 2.3 Rodent perception

Data were collected from seven adult male rats weighing 300–400 g. Long Evans rats were selected for their high visual acuity (~1.0 cycle/degree; Prusky et al., 2002). Rats were group‐housed in environmentally enriched enclosures with a 12:12 hr reversed light–dark cycle. Animals had ad libitum access to food, but daily water consumption was restricted to rewards obtained during experimentation as well as a 2‐hr period of ad libitum access following the last test session in a day. Test sessions were run once or twice daily, 5 days/week. On nontesting days, animals had ad libitum access to water. The training period ranged from 52 to 106 testing days.

Three rats of an initial cohort of 10 were excluded due to unavoidable time constraints that prevented the completion of their training.

### 2.4 Testing apparatus

Rodents were trained and tested in a custom Plexiglas chamber (20 W × 30 L × 40 H cm) with two beam‐break detectors (Little Bird Electronics, GP1A57HRJ00F) embedded in the front “viewing” wall of the enclosure. To activate the sensors, rats blocked the infrared beam with their nose. The rats initiated stimulus presentation by activating the central sensor and reported their percept by leaving the central sensor and activating the flanking sensor, which incorporated a 16‐gauge stainless steel tube for reward delivery from a computer‐controlled syringe pump (New Era Pump Systems, NE‐500). Visual stimuli were presented to rats on 120 Hz LCD monitors (Samsung 2232RZ or Eizo FG2421; Ghodrati, Morris, & Price, 2015) positioned 25 cm from the viewing wall. All stimuli were generated in MATLAB, using the Psychophysics Toolbox extensions (Brainard, 1997; Kleiner, Brainard, & Pelli, 2007; Pelli, 1997).

Custom MATLAB scripts were used to sample the photo‐interrupter outputs at 120 Hz (Measurement Computing, USB 1208FS), register rat behavior, control stimulus presentation, and administer rewards or timeouts.

### 2.5 Stimulus details

**Figure 1:**
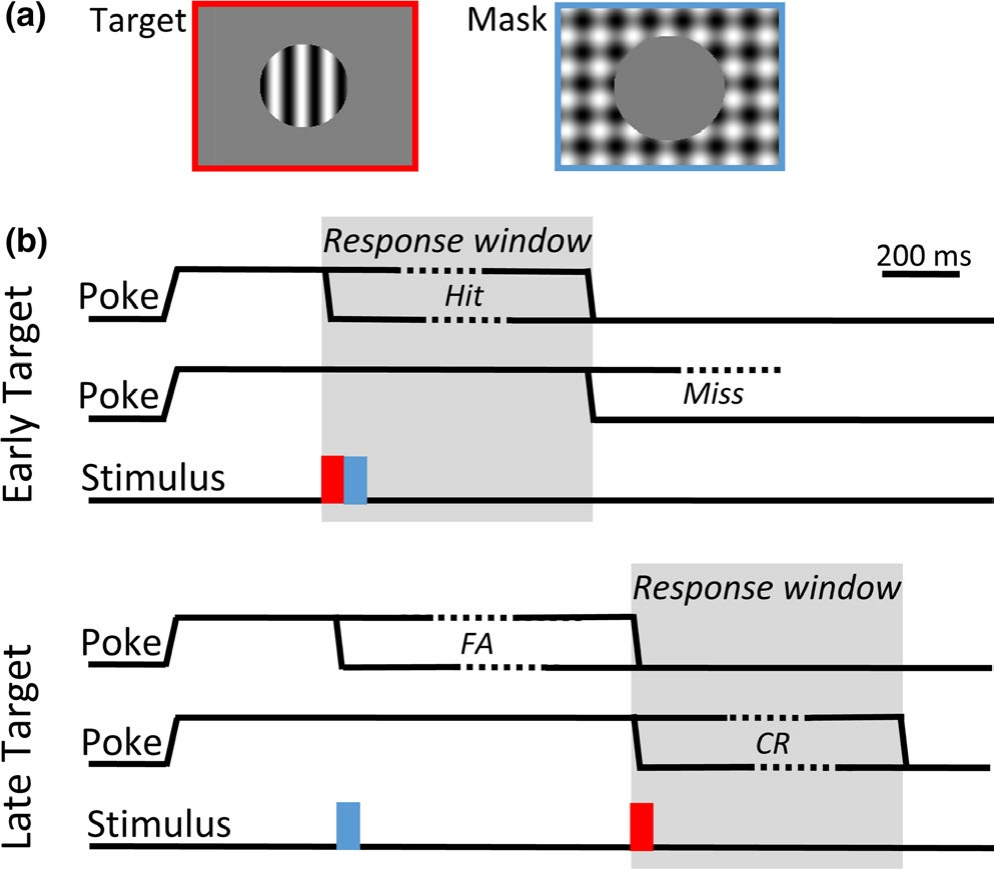
Stimulus and Task schematic. (a) Target stimuli were sine‐wave gratings with orientation 0 or 90°, spatial frequency 0.1 cpd, and presented in 51° diameter aperture. Mask stimuli were a full‐screen plaid (91 by 58.5°) created by the sum of both target orientations, with a 56° aperture centered over the target location. Colored boxes are for highlighting purposes and were not shown to the animals. (b) Following a central nose poke, either an Early Target (400 ms) or Late Target (1,200/1,300 ms) was presented. From the onset of the target, the rats had 700 ms (rats 1, 2, 4 and 5) or 800 ms (rats 3, 6 and 7) *Response window* to exit the central sensor and then a further 2 s to enter the flanking sensor to report their detection. Mask stimuli were presented in 67% of trials at a 450 ms delay from the onset of the trial. Target stimuli presented in the early interval varied in contrast between 6.25%–100%. Trials with a late target onset served as catch trials, but included a 100% contrast target to monitor the animal's motivation. Hit and miss rates were calculated from trials with an early target onset. Correct reject and false alarm rates were calculated from trials with a late target onset. Correct rejects required animals to withhold a response during the early period, and then respond to the high contrast target in the late period

Target stimuli were sine‐wave gratings with orientation 0 or 90°, spatial frequency 0.1 cpd, and presented in a circular aperture with diameter 51°. Mask stimuli were a full‐screen plaid (91 by 58.5°) created by the sum of both target orientations, but with a 56° aperture centered over the target location. Thus, there was a 2.5° separation between the outside edge of the target and the inside edge of the mask aperture (Figure 1a). Target stimuli were presented for 48 ms at one of four contrasts (12.5%, 25%, 50% and 100%). Two animals (rats 2 and 3) reliably detected the 12.5% contrast and thus an additional 6.25% condition was also included for these animals. The duration of the mask was 72 ms, and the contrast was held constant throughout the testing period at either 50% (rats 1, 4, 6 and 7) or 100% (rats 2, 3 and 5), depending on the capability of the animal (Table 1). If rats were unable to reach our threshold criterion of 70% correct detection of high contrast targets in the presence of a 100% contrast mask, they proceeded into the task with the 50% mask contrast.

**Table 1:** Task parameters were adjusted for each rat according to their performance and response time capabilities

Rat No.	Mask contrast (%)	Response window (ms)	Late target onset delay (ms)
1	50	700	1,200
2	100	700	1,200
3	100	800	1,300
4	50	700	1,200
5	100	700	1,200
6	50	800	1,300
7	50	800	1,300

### 2.6 Rodent task design

Rodents were trained to perform a two‐interval detection task, in which they were rewarded for correctly detecting a target grating that varied in contrast and that was sometimes followed by a mask stimulus. Rats initiated a trial by blocking the infrared beam of the central sensor with their nose and reported target detections by leaving the central sensor and activating the flanking report sensor. Once a trial was initiated, if the rat maintained the central nose poke, a target stimulus was presented at either an early (400 ms), or late (1,200/1,300 ms) delay from the onset of the trial (Figure 1b). The allowed response window for rats to exit the central sensor always began at the onset of the target stimulus. In order to determine if the rats were reporting the presence of an early target, the early and late response windows could not overlap in time. We also added a 100 ms gap between the two possible response windows. Initially, we trained animals with a 700 ms response window, meaning that the late target could not be presented earlier than 1,200 ms after the initiating nose poke (400 + 700 + 100 ms). However, some animals had slower response times during the early training phase, so we allowed an 800 ms response window and a corresponding 1,300 ms delay. Rats were given an additional 2 s from exiting the central sensor to enter the flanking sensor to report their detection and collect their reward.

Two‐thirds of trials included a mask stimulus, which was presented at a delay of 450 ms from the onset of the trial. Thus, there was a 50 ms SOA between the early target (if it appeared) and the mask. Early targets had variable contrast (6.25%–100%) but targets presented in the late interval always had 100% contrast and no mask stimulus. The easily detectable late stimulus thus provided an efficient method to reward animals' correct rejection behavior in the absence of the early stimulus. In this way, there were four trial categories enabling us to monitor numerous aspects of the rodent behavior: (a) early target‐only trials, which enabled us to observe the rodents' ability to detect each target contrast in the absence of a mask; (b) early target + mask trials which enabled us to investigate the effect of a mask on target detection; (c) late target‐only trials which enabled us to monitor the proportion of false detection trials, in which the rats responded in the early window, regardless of stimulus presentation; and (d) early mask + late target trials, which allowed us to determine the rate of incorrect responses to the mask.

On each trial, the rats had 700/800 ms from the onset of the target to exit the central sensor and then 2 s to activate the flanking report sensor to indicate their detection. Any exits from the central sensor that were not followed by a nosepoke at the report sensor were ignored and excluded from analyses. Correct responses were rewarded with 75–175 μl of 5% sucrose solution. To encourage rats to perform the task correctly, we implemented a ramped reward system, in which the reward volume increased with each consecutively correct trial: The first correct response following an error received 75 μl; two consecutive correct responses received 100 μl; and three or more consecutive correct answers received 175 μl. Incorrect nose pokes at the flanking sensor received no reward and triggered a brief 3.3 kHz error tone. To discourage rats from exiting the central sensor prematurely, a new trial could not be initiated before 2.9–3.1 s had passed since the start of the previous trial.

To prevent rats from developing a time‐dependent response strategy, if rats made two consecutive incorrect choices for the same target delay, a “correction trial” was implemented, in which the target delay was fixed until a correct response was obtained. All correction trials were excluded from analyses. On average, for the final task design, correction trials represented <10 percent of the trials completed.

### 2.7 Training procedure

Altogether, it took 52–106 training days (with 1–2 sessions/day) to shape each animal's behavior for the final task. Seven phases of training were used to shape behavior. These phases and the approximate number of training sessions required to progress were: (a) nosepoke‐reward association (four sessions); (b) central then side nosepokes to receive reward (four sessions); (c) patience training, so they waited up to 1,350 ms before seeing a visual stimulus that indicated they could receive a reward at the side nosepokes (30–74 sessions); (d) respond to small, brief targets (4–12 sessions, but rats 3 and 5 required 42 and 26 sessions); (e) two interval training, so the target stimulus could appear either early or late (4–26 sessions); (f) mask introduction (48–70 sessions); and (g) variable target contrast (6–12 sessions). The criterion to progress from one phase to the next was 70% correct in two consecutive sessions.

The most challenging aspect of training was the introduction of the mask. Initially, we started with low‐contrast masks that were spatially separated from the border of the target. When animals reached criterion performance (70%, as above), contrast was increased, usually in 10% steps, and then the separation of the target and mask was decreased. With this method, three rats were unable to reach criterion to progress to a 100% contrast mask and were therefore tested at 50%.

### 2.8 Analyses

#### 2.8.1 Trial exclusion

After animals had reached threshold performance, test sessions were excluded from analyses if the rats incorrectly responded to the mask in the absence of a target at a rate more than two standard deviations above their average performance across all sessions. This resulted in the exclusion of a maximum of two data sessions (~400–600 trials) per animal. Correction trials and trials where the rats did not activate the flanking sensor were excluded from all analyses. Altogether, we collected 3,340–8,768 valid trials across 25–38 sessions per animal. We found no trends or significant differences between performance across the 5 testing days within a week (animals had free access to water on weekends) or between the morning and afternoon testing sessions. Therefore, data from all testing sessions were combined. Full details of the training and test process, and individual performance trajectories for each animal are described in (Richards, 2017).

#### 2.8.2 Detection and response bias calculations

We quantified target detectability for each contrast in both target‐only and masked trials using the sensitivity index (*d*′), a statistic used in signal detection theory to measure the separation between noise and signal distributions: *d*′ = *z* (Hit rate)−*z* (False alarm rate), where *z* (*X*) indicates the *z*‐score of the proportion *X*. Note that we assume the underlying signal and noise distributions are Gaussian. This assumption can be problematic if bias is strong, meaning that the hit or false alarm rates are close to 0 or 1, but this was not the case in our data sets.

## 3 RESULTS

### 3.1 Human detection performance is impaired by the presence of a mask

**Figure 2:**
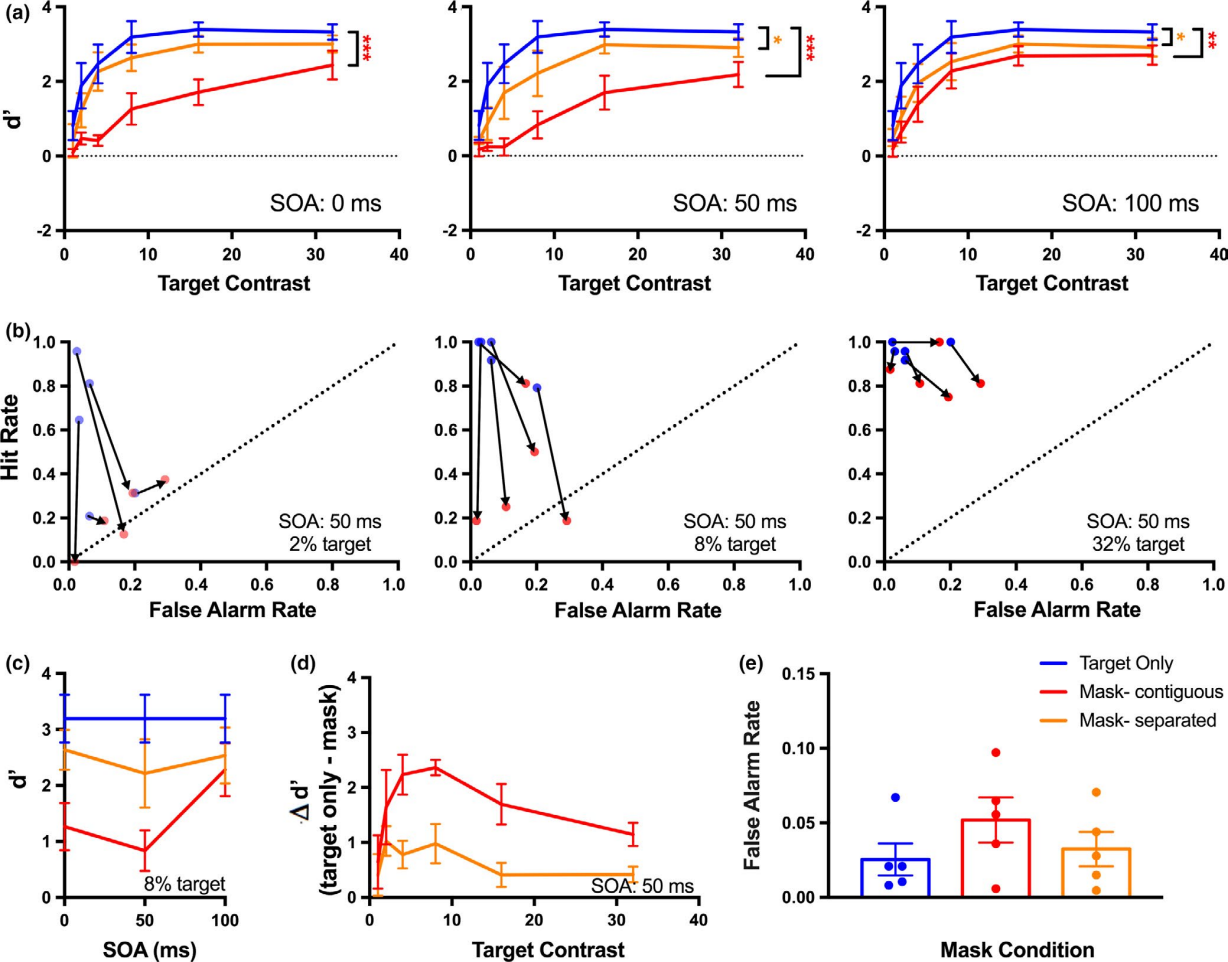
Human detection performance is impaired by the presentation of a mask. (a) Detection performance (*d*′) was measured for a target‐only, and two masked conditions, across a range of target contrasts. Results show mean (±*SE*) for five participants. The masks were presented at three SOAs: 0, 50, and 100 ms. The target‐only condition is the same dataset for each SOA. (b) For each participant, the changes in hit and false alarm rates from target‐only to the contiguous masked trials are shown for three target contrasts (2%, 8%, 32%) in the 50 ms SOA condition. Arrows connect the target‐only and masked data for each participant. (c) Detection performance for the 8% target contrast plotted across SOA demonstrates a U‐shape function for both mask conditions. (d) The difference in target detection accuracy (*d*′) between target‐only and masked trials is shown for the 50 ms SOA condition. The effect of the mask was greatest when the target was closer to the contrast detection threshold. (e) False alarm rates were generally low and not significantly affected by the mask condition. Points show individual subject data. (***) *p* < .0001; (**) *p* < .01; (*) *p* < .05

We examined how the detection of a target grating was affected by target contrast, target‐mask separation, and SOA. As expected, regardless of SOA and target‐mask separation, target detectability, quantified using the dimensionless statistic *d*′, was significantly affected by contrast (Figure 2a; *p*_SOA0_ < .0001, *F*_5,20_ = 56.17; *p*_SOA50_ < .0001, *F*_5,20_ = 30.33; *p*_SOA100_ < .0001, *F*_5,20_ = 26.4; two‐way ANOVA).

The presence of a mask reduced target detectability relative to the performance in target‐only trials, and the contiguous mask produced larger reductions in *d*′ than the spatially separated mask. The presence of a mask, regardless of its separation from the target in space or time, caused a large reduction in the hit rate as well as a small increase in the false alarm rate for each target contrast (Figure 2b). For both the contiguous and separated masks, the reduction in *d*′ was greatest for the 50 ms SOA condition, indicating a U‐shaped psychometric curve (type‐B masking). This was clearest for 8% contrast targets, but was evident for a range of contrasts (Figure 2c). To determine if the effect of the mask varied across contrast, we calculated the difference in target detectability between target‐only and masked trials. In general, the effect of the mask, regardless of its separation, was greatest when the contrast of the target was closer to threshold. This is shown for the 50 ms SOA where masking was most effective, but was also evident at other SOAs (Figure 2d). For each SOA, detectability was significantly affected by the mask condition (*p*_SOA0_ < .001, *F*_2,8_ = 33.59; *p*_SOA50_ < .001, *F*_2,8_ = 32.12; *p*_SOA100_ = .0015, *F*_2,8_ = 16.4; two‐way ANOVA). Post hoc analyses revealed that the presence of a contiguous mask reduced detection performance relative to the target‐only trials across all SOAs (*p*_SOA0_ < .001, *p*_SOA50_ < .001, *p*_SOA100_ = .0012; Tukey's multiple comparisons test). The effects of the spatially separated mask were only significant for the 50 and 100 ms SOA (*p*_SOA0_ = .109, *p*_SOA50_ = .0264, *p*_SOA100_ = .0216; Tukey's multiple comparisons test).

In order to determine if the presence of a mask stimulus influenced the participants' bias to respond, we calculated false alarm rates across each masked condition. In general, there was little bias to respond, however, the false alarm rate did increase in the presence of a mask, in particular for the contiguous mask condition (Figure 2e). The effect of the mask condition on the false alarm rate was not significant (*p* = .0587, *F*_2,8_ = 5.365; one‐way ANOVA).

### 3.2 Rats reliably detected targets, but were biased by masks

In order to determine if visual masking produced similar perceptual deficits in rodents to those in humans, we trained rats to perform a target detection task. Ideally, the task would explore how multiple stimulus manipulations affect visual masking (e.g., target contrast, mask contrast, SOA, size, spatial overlap, and spatial separation); however in an animal model, training on multiple parameter manipulations is difficult and time consuming. Although SOA is the most common manipulation in visual masking research, our previous investigation manipulating SOA in a 2AFC discrimination task suggested that SOA interacted with the animals' impulsiveness (Dell et al., 2018), impairing our ability to determine if perceptual masking was present. To avoid confusing the rats with multiple parameter manipulations, here, we only varied the contrast of the target and presented the mask at a fixed 50 ms SOA, as this produced the largest masking effect in humans. By using a detection task, animals only had a single behavioral response and rule for each trial type (i.e., exit the central nose poke when the target is visible), whereas in the discrimination task animals had to exit the central nose poke and move to one of two lateral nose pokes.

**Figure 3:**
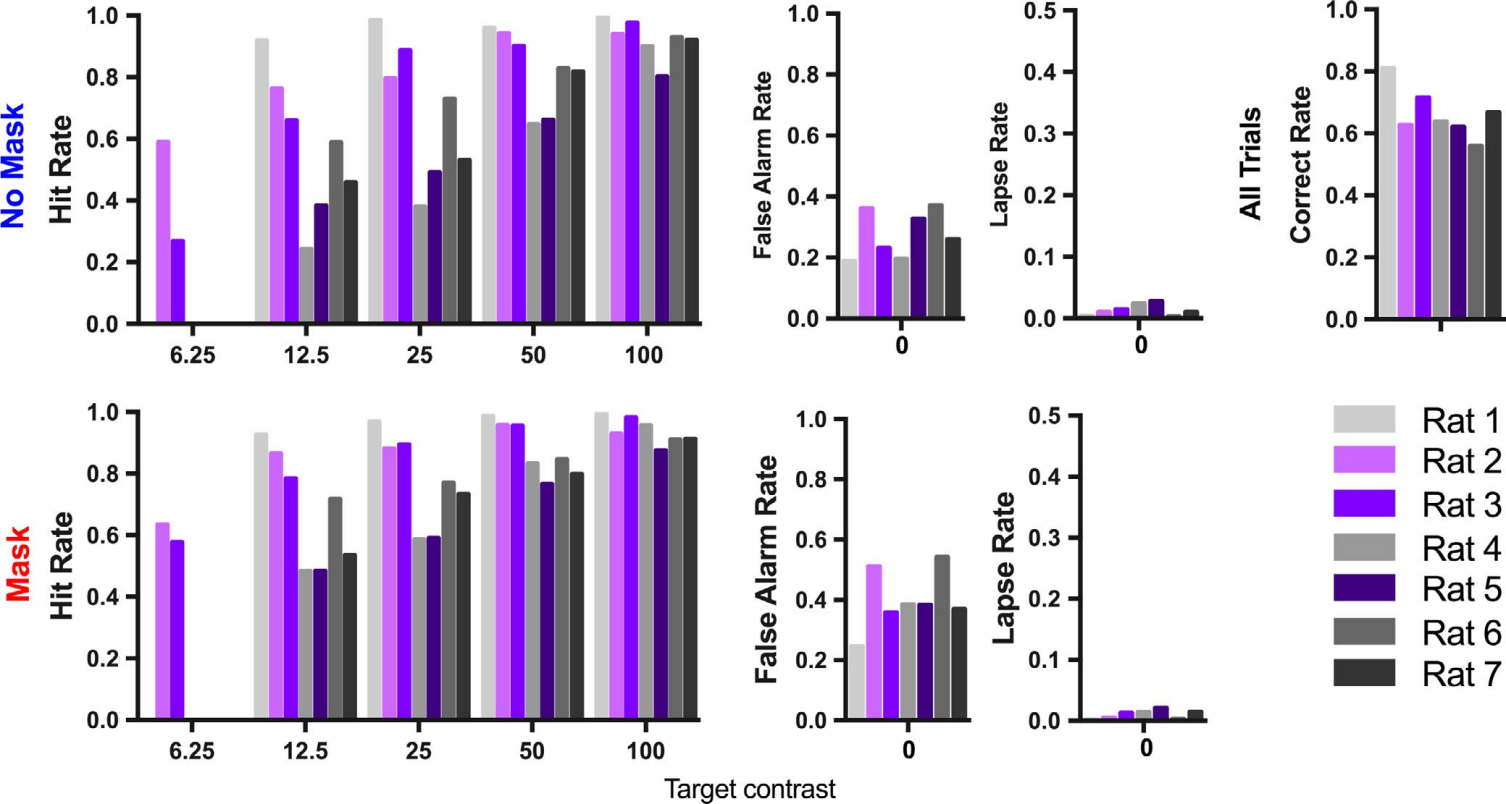
Rat detection performance is consistently contrast‐dependent across animals. Hit, false alarm, and lapse rates for trials without a mask (top panels) and with a mask (bottom panels). Correct rates calculated from all trials indicate the rate at which the rats were rewarded. Each color represents the performance of a single rat, animals shown in gray were tested with 50% contrast masks; other animals were tested with 100% contrast masks. A target stimulus was presented on every trial at either a 400 ms or 1,200/1,300 ms delay from trial onset. Only the targets that were presented in the early interval varied in contrast and were used to measure the rate of hits and misses. Target stimuli presented in the late interval were always 100% contrast and were used to calculate the false alarm rate. Only rats 2 and 3 were tested with target contrasts of 6.25%

For each animal, we calculated hit, false alarm, and lapse rates across the different contrast and mask conditions (Figure 3). Hit rates were calculated from trials with an early target while false alarm and lapse rates were calculated from late target trials. Across all trials, we also calculated the proportion of trials on which the rats were rewarded, as a measure of overall performance. Although overall performance (correct rate) ranged from just 56%–82% across animals, lapse rates were consistently low (<3%) for all animals, indicating that they had sufficient motivation to perform the task correctly. Hit rates increased with the contrast of the target, regardless of the presence/absence of the mask. However, false alarm rates were relatively high and increased with the presence of a mask. The effects of the mask on response bias will be addressed in more detail below. Given that the hit rate is subject to bias, it is not a suitable indicator of target detectability in our study, thus below, we use the metric *d*′.

### 3.3 Target detectability is affected by the contrast of the target

**Figure 4:**
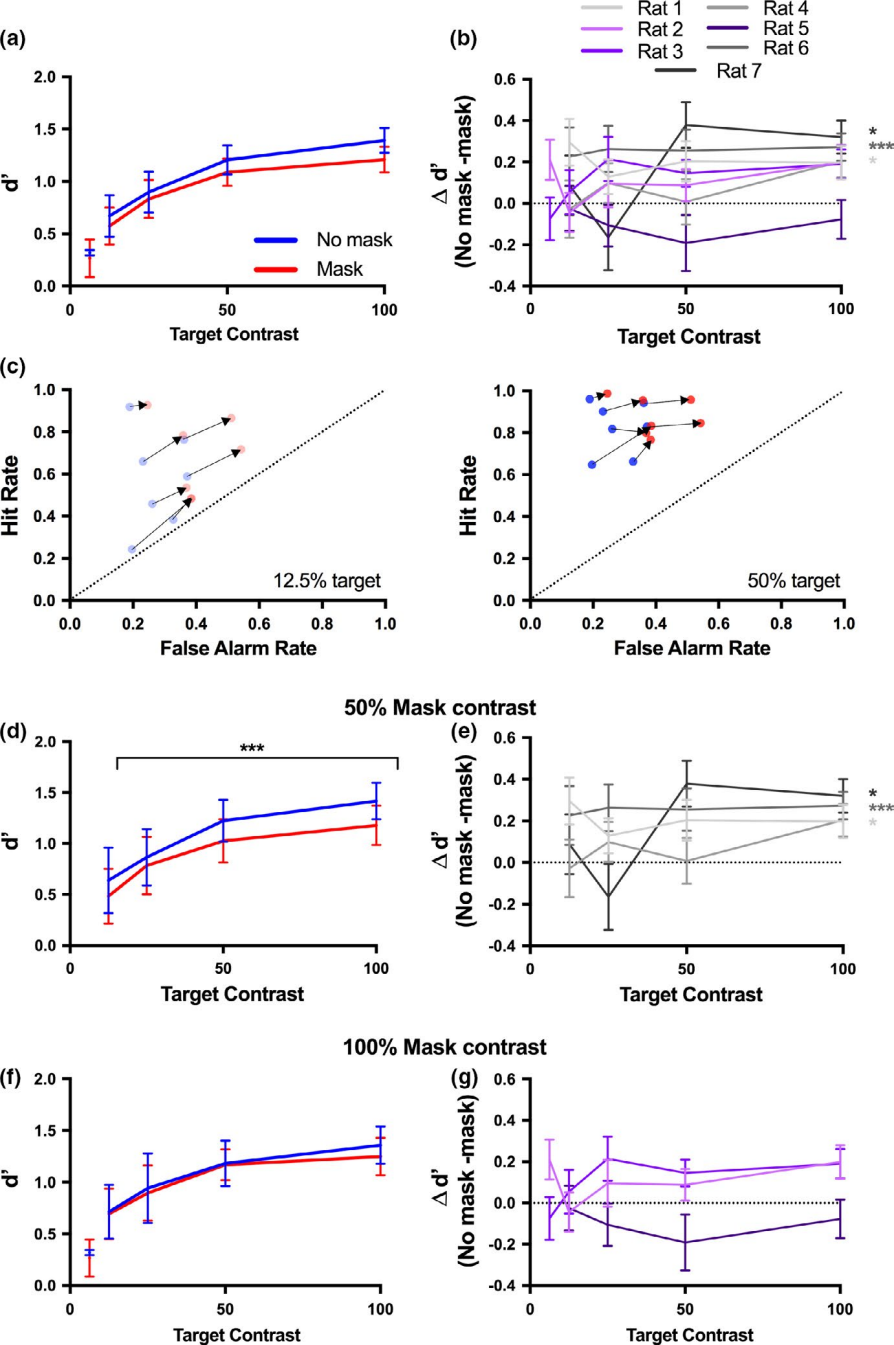
Target detectability depends on target contrast but is not consistently impaired by a mask. (a) Detection performance (*d*′) was averaged across test sessions and across animals. This is shown separately for animals performing the task with a (d) 50% and (f) 100% contrast mask. (b) The difference in target detection accuracy (*d*′) between target‐only and masked trials is represented for each animal and shown separately for animals performing the task with a (e) 50% and (g) 100% contrast mask. The positive values indicate that most animals performed better in the target‐only trials, with the exception of rat 5, who performed better in the presence of a mask. 6.25% contrast condition represents the average performance of two animals (rats 2 and 3). (c) For each rat, the change in hit and false alarm rates between target‐only and masked trials is shown for two target contrasts (12.5% and 50%). Arrows connect the target‐only and masked data for each rat. Error bars represent 1 *SE* across testing sessions (25–38 sessions/animal). *—*p* < .05; ***—*p* < .001

We examined the effects of target contrast and the presence of a mask on target detectability using *d*′, because it accounts for an animal's tendency to respond in the absence of a stimulus (false alarm rate). Note that, although rats performed trials with either a 50% or 100% contrast mask, our training results demonstrated that each animal was performing the task near their psychophysical threshold. As in the human data, we found that *d*′ significantly increased with target contrast regardless of if a mask were present (Figure 4a; *p*_Contrast_ < .001, *F*_3,48_ = 6.972). The effect of target contrast on detectability was significant for all animals (*p*_Rat1_ < .0001, *F*_3,75_ = 11.7; *p*_Rat2_ < .0001 *F*_4,120_ = 59.89; *p*_Rat3_ < .0001, *F*_4,144_ = 144.6; *p*_Rat4_ < .0001, *F*_3,93_ = 104.6; *p*_Rat5_ < .0001, *F*_3,102_ = 88.51; *p*_Rat6_ < .001, *F*_3,93_ = 28.4; *p*_Rat7_ < .0001, *F*_3,72_ = 51.75; two‐way ANOVA).

If visual masking impaired target perception, we would expect that *d*′ would be lower in masked trials compared with target‐only trials. While on average this was true, there was no statistically significant effect of the mask on target detectability across the population (Figure 4a; *p*_Mask_ = .3128, *F*_1,48_ = 1.04). In general, individual animals performed worse in masked trials across all target contrasts, as indicated by the positive values in Figure 4b. However, this trend was significant for only three of seven animals (*p*_Rat1_ = .013, *F*_1,25_ = 7.19; *p*_Rat2_ = .14, *F*_1,30_ = 2.349; *p*_Rat3_ = .11, *F*_1,36_ = 2.745; *p*_Rat4_ = .33, *F*_1,31_ = 0.97; *p*_Rat5_ = .17, *F*_1,34_ = 1.95; *p*_Rat6_ = .001, *F*_1,31_ = 13.28; *p*_Rat7_ = .048, *F*_1,24_ = 4.30; two‐way ANOVA). Unlike in humans, we did not see any systematic change in the effect of the mask across target contrasts. The presence of the mask did not reduce the hit rates for the rats, instead, it increased both hit and false alarm rates across all target contrasts (Figure 4c).

Given that the strength of a mask tends to increase with its contrast (Breitmeyer & Ogmen, 2006), we were interested to see if the effect of the mask was greater for the rats performing the task with a 100% contrast mask. However, counter to expectations, we found that the three rats whose performance was significantly impaired by the presence of a mask were all performing the task with a 50% contrast mask (Figure 4d,e; rats 1, 4, 6 and 7; stats reported above). Rats performing the task with a 100% contrast mask were not significantly impaired by its presence, although two of these animals still tended to perform worse in the presence of the mask (Figure 4f,G; rats 2, 3 and 5; stats reported above).

To determine if the animals that were significantly affected by the presence of a mask were related in any other aspect of the task or their behavior, we examined the response window duration (700 vs. 800 ms); their overall performance; their false alarm rates; the duration of their training; and the number of sessions and trials they completed in the final task. We found no relationship between any of these factors and the effect of the mask on their behavior.

Altogether our results suggest that target detectability is affected by target contrast in a similar manner to that which occurs in humans. However, the effects of the mask on target detectability were inconsistent, with performance being significantly impaired in only three of seven animals. The difference in the effects of the mask between animals appears to correlate with the contrast of the mask. However, the animals performing the task with a 50% contrast mask were doing so because they were unable to reach criterion to progress into the final task with a 100% contrast mask. It is therefore possible that the effects of the mask differed between animals' due to differences in the animals' perceptual/cognitive capabilities rather than the contrast of the mask.

### 3.4 Uninformative mask stimuli increase the bias to respond

**Figure 5:**
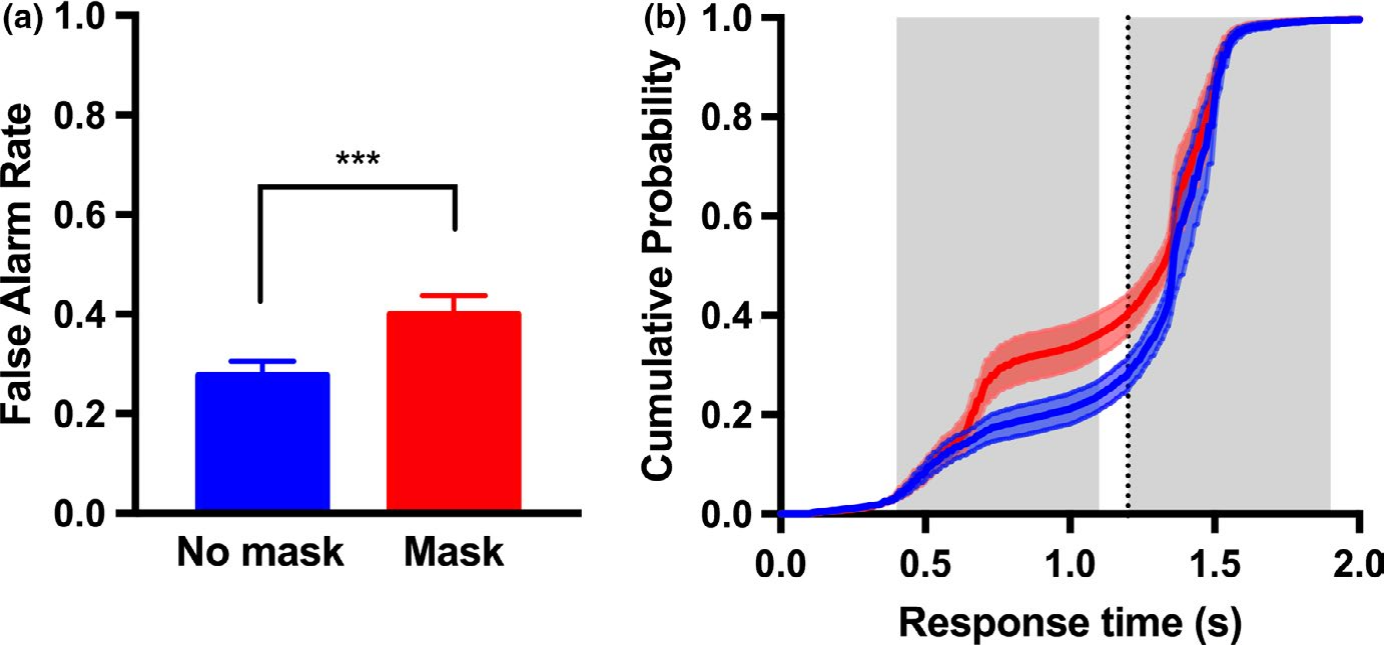
Rodents respond impulsively and are biased to respond in the presence of a mask. (a) Response bias measured as the false alarm rate was averaged across animals (*n* = 7). Animals were biased to respond and this bias was exacerbated by the presentation of a mask. (b) The cumulative probability of response over time was averaged across animals for late target trials. The gray shaded windows indicate the early and late target response windows. The vertical dotted line indicates the onset of the late target. Error bars represent 1 *SE*. (***) *p* < .001

In a Go/No‐Go detection task, animals are commonly rewarded for responding (e.g., licking) when a target stimulus is present, and withholding the response when a target is absent. Therefore, impulsivity or a bias to respond (lick) will increase the hit rate, at the expense of increased false alarms. Given our previous observations in the discrimination task that rats respond impulsively, often failing to wait for visual cues, we specifically included trials in which the target was absent in the early interval. In these trials, animals were required to respond to a high contrast target that was presented in the late interval. This allowed us to monitor the rate of false alarm responses in the absence of a target stimulus, and critically, how the presence of a mask impacted this response bias. In signal detection theory, response bias is traditionally calculated as the criterion *c* = *z* (hit rate) + *z* (false alarm rate)/2. However, given that our noise distribution (false alarm rate) was the same for each target contrast, whereas the hit rate necessarily changes with contrast, the criterion metric does not provide any additional information compared to the false alarm rate on its own. We therefore quantified the effect of the mask on response bias by comparing the rate of false alarms in late target trials in which a mask was present or absent during the early window. Rats produced a false alarm (i.e., responded before or during the early response window, in the absence of a stimulus), in 28% of the late target trials (Figure 5a). The presentation of a mask significantly increased the average rate of false alarms by 12% (Figure 5a; *p* < .001, *t*_6_ = 6.175; paired *t* test). Altogether this indicates that while the animals were biased to respond early, the mask exacerbated this bias.

In Figure 5b, we show the cumulative probability of response throughout the duration of a trial, for the late target‐only and late target + early mask conditions. In the absence of any visual stimulus (blue trace), rats tended to respond impulsively within the early response window, and notably, at times that were consistent with when an early target would have appeared. In the presence of a mask (but with no target), rats responded more frequently in the early response window, in a manner that was time‐locked to the appearance of the mask (divergence of red and blue traces). This suggests that the rats used trial timing cues to respond.

### 3.5 Rats' responses are primarily visually evoked

**Figure 6:**
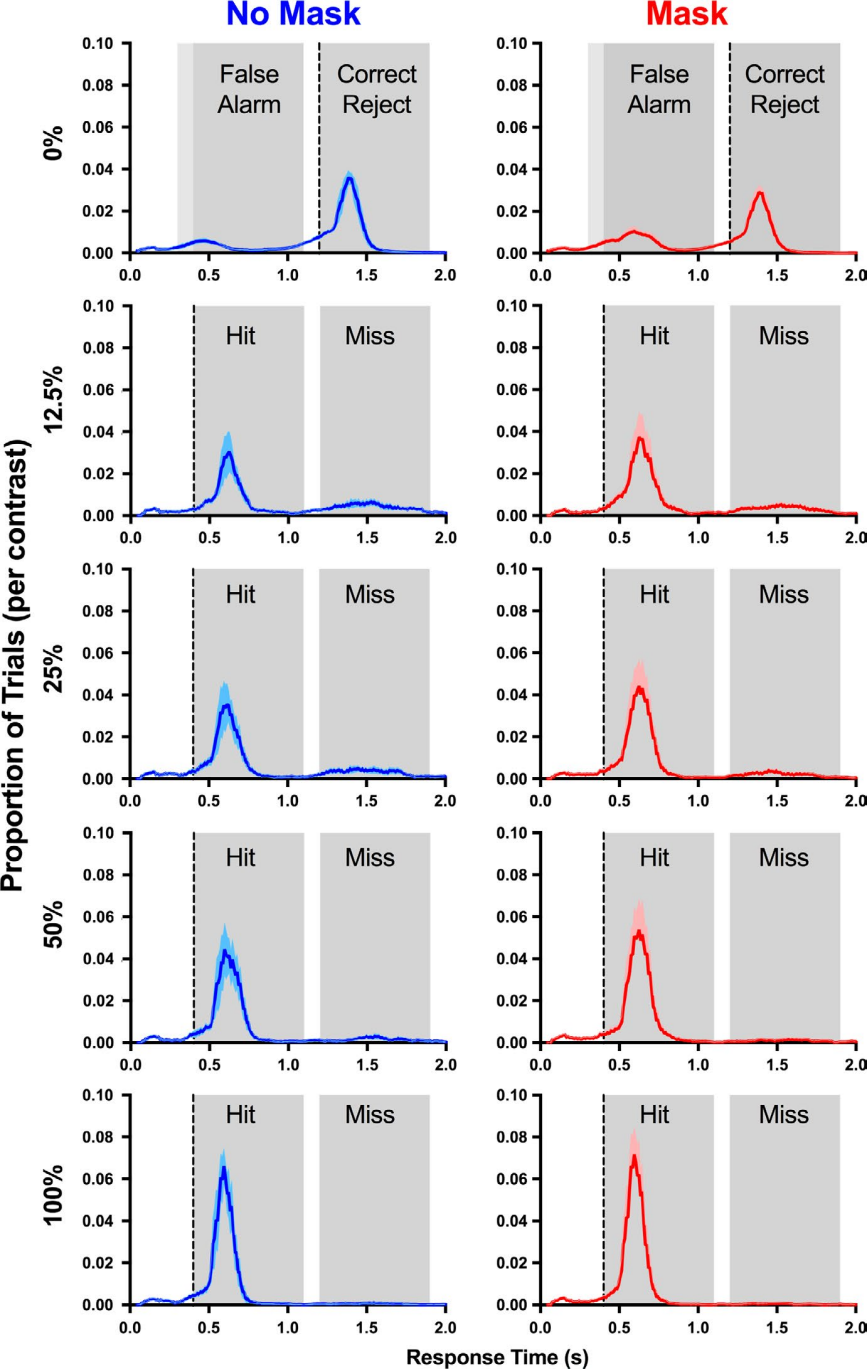
Rodent responses are predominantly visually driven. The distributions of response times were aligned according to the onset of the target stimulus (vertical dotted line) and averaged across animals (*n* = 7). The gray shaded windows illustrate the early interval and late interval response windows. The 0% target condition represents the trials with a late target, where there was no target presented in the early interval but a 100% target presented in the late interval. Note that for this condition the response times were aligned to the late target onset, meaning that animals expected the early response window to begin either 800 or 900 ms prior to the late target onset depending on the late target delay. Response times were calculated from the onset of the early target until the time the rats exited the central sensor. Only trials where the rat activated the flanking report sensor within 2 s of exiting the central sensor were included in these analyses. The red and blue shaded regions represent mean (*SE*)

Given the possibility that the rats were responding in a timing dependent manner, we specifically analyzed when the rats were exiting the central sensor to report their detection (Figure 6). In general, the rats tended to respond within one of the two possible response windows. When they missed an early, low‐contrast target they were most likely to respond within the first few hundred milliseconds of the late response window (just after the late target should have appeared). In the late target trials, the rats rarely missed the target (see lapse rates; Figure 2), and any false alarms tended to occur within the early response window. Collectively, this indicates that the rats understood that rewards could be obtained by responding in one of two response windows. However, regardless of the target contrast or the presence of a mask, the rats were most likely to respond shortly after target presentation. There was a narrow distribution of response times for both hits and correct rejects, in fact, for the 100% contrast early target, more than 80% of responses occurred within a 200 ms period beginning 100 ms after the target onset. This suggests that the rats sometimes responded according to a timing cue, but in most cases correctly responded to the target stimulus.

### 3.6 Response times are faster for high target contrasts

**Figure 7:**
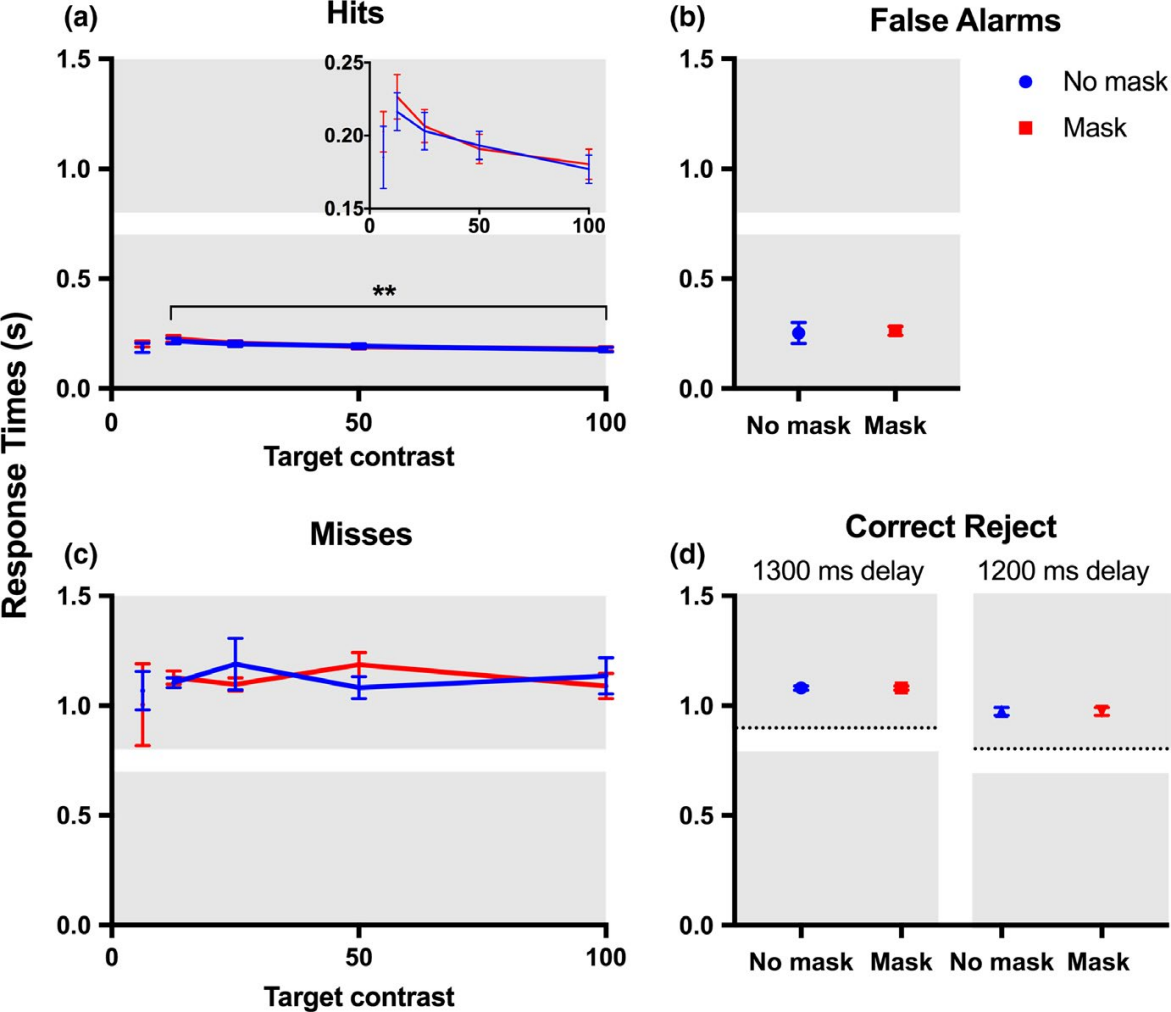
Response times are affected by target contrast. Response times were measured from the time of target onset in the early interval, regardless of whether the target was presented in the early or late interval. The median response times were averaged across animals (*n* = 7) for (a) hit, (b) false alarm, (c) miss, and (d) correct reject trials. The inset in panel (a) shows a zoomed axis of the effect of target contrast on response times in the hit trials. Incorrect responses where the rodents (c) missed the target or (b) responded in the absence of a target tended to fall within the incorrect response window. Trials were considered a correct reject when the animal correctly withheld a response during the early interval and then correctly responded to the 100% contrast target presented in the late interval. Late targets were presented at either a 1,200 ms delay from trial onset (rats 1, 2, 4 and 5) or a 1,300 ms delay (rats 3, 6 and 7). Response times for the 6.25% contrast condition represent an average of only 2 animals (rat 2 and 3). The dotted lines in plot (d) represent the late target onset, and the gray boxes illustrate the response windows for the early and late intervals. Error bars represent ± *SE*. (**) *p* < .01

To further understand how the rat response times were affected by the target and mask stimuli, for each response type (hit, miss, false alarm, and correct reject), we calculated response times from the onset of the early target to the time that the rats exited the central sensor. In general, response times were less variable when the animals responded correctly (Figure 7a,b; hits and correct rejects) when compared with incorrect responses (Figure 7c,d; misses and false alarms). For hit trials, the response times were influenced by target contrast (*p*_Hit_ < .01, *F*_3,48_ = 4.889; two‐way ANOVA), with response times becoming significantly shorter at the higher target contrasts. However, response times were not significantly affected by target contrast in miss trials, with the response times remaining approximately the same across all target contrasts (*p*_Miss_ = .9587, *F*_3,42_ = 0.1016; two‐way ANOVA). These results suggest that in correct trials, the animals were responding to the target stimulus and were thus affected by the contrast of the target, while in the incorrect trials, the animals were responding independently of the target stimulus, presumably in a time‐dependent manner. To further explore this possibility, we compared the distribution of response times between hits with the lowest contrast tested for all animals (12.5%) and false alarm responses. We expected that if rats were responding according to a timing cue in the incorrect trials, the response times would be similar between these groups. We found this to be true; there were no significant differences between hit and false alarm response times (*p* = .2742, *F*_1,6_ = 1.448; two‐way ANOVA). In all response types, there were no significant effects of the mask on response times (*p*_Hit_ = .6638, *F*_1,48_ = 0.1914; *p*_Miss_ = .9630, *F*_1,42_ = 0.0021; two‐way ANOVA; *p*_CorrectReject_1300_ = 1.00, *t*_4_ = .003; *p*_CorrectReject_1200_ = .99, *t*_6_ = .007; *p*_FalseAlarm_ = .86, *t*_12_ = .186; paired *t* test).

## 4 DISCUSSION

We sought to determine if perceptual masking was present in rats performing a detection task. In humans, we found that detection performance systematically improved with increasing target contrast and was reduced by the presence of a mask. In order to determine if similar perceptual deficits occurred in a rodent species, we trained rats to perform a two‐interval detection task. Similar to our human data, rodent detection performance was significantly affected by the contrast of the target and was generally reduced by the presence of a mask. However, the effect of the mask on target detection was only significant for three of seven animals, all of which were performing the task with a 50% contrast mask. Counter‐intuitively, rats performing the task with a 100% contrast mask were unaffected by its presence. Below we discuss: (a) the possible influence of mask contrast on rodent behavior; (b) the limitations of a rodent model for research in visual masking and perception; and (c) the differences between rodent and human perception that may have affected our results.

Given that previous perceptual studies have shown that the strength of a mask increases with its contrast (Breitmeyer & Ogmen, 2006; Weisstein, 1972), we expected that the effect of the mask on rodent perception would be greater for animals performing the task with a 100% contrast mask. Counter to this, we only found a significant effect of the mask on target detection in 3 of 4 rats performing the task with a 50% contrast mask. The difference in the effect of the mask across the population could not be attributed to any other aspect of the animal's behavior or training. Although unexpected, this result is not unprecedented given that the shape of the trend across SOA can depend on the relative energy (contrast × duration × size) of the target and mask stimuli (Breitmeyer & Ogmen, 2006). For example, in humans the peak masking effect has been shown to shift from an SOA of 56–36 ms when the duration of the mask was increased from 2 to 32 ms (Breitmeyer, 1978). Similarly, the peak masking effect is expected to shift to shorter SOAs as the contrast of the mask is increased (Francis, 2003). In this way, it is possible that the peak masking effect shifted with the contrast of the mask so that perceptual deficits were only evident at an SOA of 50 ms when the contrast of the mask was 50%. Unfortunately, it is impossible to determine whether this was actually the case without additional data from another SOA or from multiple mask contrasts within the same animal. However, interleaving additional SOA conditions can make animal testing more complicated because of their strong tendency to respond impulsively.

We note that the animals performing the task with a 50% contrast mask were doing so because they were unable to reach our criterion (70% correct) to progress into the final task with a 100% contrast mask. The changes in target detection that we observed in these animals may therefore reflect the same behavioral limitations that prevented the animals from being able to perform the task with a 100% contrast mask in the first place. At the very least, the difference in the learning capabilities that we observed between rats implies there may be significant limitations for studying complex visually driven behaviors in rodents.

Rodents have become a popular choice for visual research; however, it is clear that they are not a perfect model of human visual processing and perception. The effect of contrast on stimulus detection provides a good example of when the psychophysical results in rodents closely replicate that of humans, albeit with greatly increased contrast detection thresholds (Busse et al., 2011; Histed et al., 2012). However, the perceptual effects of contextual modulation are often different between species. Here, masking effects were only significant in three rats and did not systematically change with target contrast, as they did in humans. Similarly, the influence of spatial context on target perception has been shown to be different between rats and humans, where, despite controlling for stimuli, task, and learning procedures, the presentation of collinear flankers enhanced target detection in humans but impaired detection in rats (Meier & Reinagel, 2013). Oculomotor behaviors such as smooth tracking and foveating saccades are also clearly different between rodents and humans. However, whereas differences in oculomotor behaviors can easily be attributed to differences in retinal structure, it is less clear what might account for differences in masking and flanking effects.

Behavioral performance is always affected by the amalgamation of sensory, and nonsensory factors. In rodents, the tendency to respond impulsively can be a particularly dominant nonsensory factor (Busse et al., 2011; Schwarz et al., 2010). In our study, we found that responses were clearly visually driven, but that the rats had a strong prior about when the stimulus should occur. Thus, impulsive responses (i.e., in the absence of a target), most often, occurred in the early response window. This suggests that the animals were highly sensitive to trial timing cues and had a preference for shorter duration trials. That is despite the fact that the target stimulus was presented in the late interval in the majority of trials (66%) and total trial duration was fixed. Thus, while impulsive responses were made at strategic times within the trial, their timing‐based strategy favoured speed over optimal reward acquisition across a testing session. This is similar to the findings of a motion discrimination task, where rat responses were predominantly governed by the time that had elapsed from the onset of the trial, rather than a criterion for evidence accumulation (Reinagel, 2013). Together, these results demonstrate that rodent behavior can be strongly influenced by trial timing cues. The influence of such nonsensory factors is difficult to avoid in an animal task, but should be considered in task design and ideally monitored throughout data acquisition. A simple solution is to remove or minimize timing contingencies in the task, so that animals can always respond at a fixed time after stimulus or trial onset.

Although rodents provide a valuable model for visual research, it is important to consider the limitations of their behavioral capabilities. Our investigations of visual masking have demonstrated that rats are capable of learning and performing complex visual tasks. Our task design is similar to 2‐interval, 2‐alternative forced choice designs used in humans (i.e., in which the two intervals contain signal, or signal + noise). We adopted this design because it is useful for over‐riding biases, but it is cognitively more challenging for the subjects, and we are not aware of previous adoption of 2I‐2AFC tasks with rats. While all our rats were able to detect target stimuli with high‐performance levels in the presence of a 50% contrast mask, more than half were unable to reach criterion to perform the final task with a 100% contrast mask. In general, the presentation of a mask significantly increased the animals' bias to respond. A similar issue was reported for a detection task in which rats were required to report the presence or absence of a target Gabor that was sometimes presented between two flanking Gabors. In that study, some rats were unable to reach adequate target detection performance when the contrast of the flankers exceeded 40% (Meier et al., 2011). Further, flankers biased the rats to report the presence of a target, and the bias increased with flanker contrast (Meier & Reinagel, 2011). These findings suggest that rat behavior may be limited in ways that impact the feasibility of some visual investigations. For example, should we have found that masking was only evident with a 100% contrast mask (and absent with the 50% contrast mask), more than half of our animals would not have been capable of performing the task with the stimulus parameters necessary to produce perceptual masking.

A related challenge is that small effect sizes demand large numbers of trials. Here we based the data on a total of up to 8,768 trials; similar previous studies have used over 2,000–3,000 trials for each stimulus condition, requiring dozens of data collection sessions simply to demonstrate the effects under study (Meier & Reinagel, 2013; Rosselli, Alemi, Ansuini, & Zoccolan, 2015). Clearly increasing the size of the data set may help us resolve backward masking in more animals, but this also highlights that the rat preparation is far less efficient than nonhuman primate experiments, especially since single‐session data collection is more amenable to simultaneous behavioral and neural recordings. For example, studies in humans can demonstrate masking reliably in a few minutes and in nonhuman primates; Bridgeman (1980) demonstrated masking in single sessions of ~200 trials.

The greatest challenge of research in visual masking, and similar phenomena such as center‐surround interactions and flanking, is that the changes in stimulus parameters that increase the likelihood of perceptual deficits arising (i.e., higher mask contrast, shorter target duration, smaller target size, less separation between the target and mask stimuli) also reduce an observer's overall performance, and therefore the sensitivity of the data to any perceptual deficits that could be occurring (Alpern, 1953; Breitmeyer, 1978, 2008; Oğmen, Breitmeyer, & Melvin, 2003; Schiller & Smith, 1968; Weisstein, 1972). This is a particularly acute problem in animal studies, where having sufficiently high performance is necessary to maintain animal motivation, because correct responses are tied to rewards. During training, we focused on reducing the separation between stimuli and increasing the contrast of the mask to increase the likelihood of masking. The fact that four of our rats were unable to reasonably perform the task with a 100% mask contrast indicates that the task parameters were already pushing the limits of the animal's capabilities. This suggests there was little room to manipulate other stimulus parameters in a way that might increase the masking effect, such as shortening the target duration or decreasing the target size. An alternative approach might be to alternate two types of testing sessions, either between days, or across weeks. This would allow stable contrast thresholds to be determined in the absence of a mask (e.g., Figure 4a). Subsequently, in a separate block of sessions, only slightly suprathreshold contrast test stimuli could be presented, with and without the presence of masks. This avoids the potentially confusing manipulation of both target and mask contrast within a single experimental session. It also ensures that in a single session, many repetitions of identical stimuli will be repeated, which is advantageous for experiments that combine electrophysiology and behavior. A complementary solution would be to use a single‐interval Yes–No design, in which observers use different actions or nosepokes to report the presence or absence of the target. This allows targets and masks to always be presented at the same time within a trial, with the considerable benefit of eliminating timing differences between trial types. However, it places a considerable burden on animals to overcome their bias to respond on target‐absent, mask‐present trials. Indeed, if all trial types are equally likely (i.e., equal proportions of trials have targets and masks present and absent) then following the erroneous rule of responding Yes to any visual stimulus (target or mask) would yield a reward rate of 75%. We believe that by separating the timing of trials leading to hits/misses and correct rejects our task design helps overcome this bias by making it more clear to animals whether they have failed to receive a reward because they missed a target (that was presented) or responded to a mask (in the absence of a target).

We have shown that rats were capable of learning and performing a complex visual masking detection task and that their ability to detect target stimuli was affected by the contrast of the target in a similar manner to that of humans. We believe that the changes in the testing method described above would increase our effect size and improve the experiment's sensitivity to reliably detect the effect of masking on behavior in individual animals. Ultimately, this will be necessary in order to determine the physiological mechanisms underlying masking, which require combining behavioral experiments with electrophysiology or imaging.

## CONFLICT OF INTEREST

None declared.

## Data Availability

The data that support the findings of this study are available from the corresponding author upon reasonable request.
